# Bioprospection of Basidiomycetes and molecular phylogenetic analysis using internal transcribed spacer (ITS) and 5.8S rRNA gene sequence

**DOI:** 10.1038/s41598-018-29046-w

**Published:** 2018-07-16

**Authors:** Thangamalai Mowna Sundari, A. Alwin Prem Anand, Packiaraj Jenifer, Rajaiah Shenbagarathai

**Affiliations:** 1 DBT - BIF centre, Lady Doak College, Madurai, 625 002 Tamilnadu India; 2 Department of Biotechnology, Lady Doak College, Madurai, 625 002 Tamilnadu India; 3 PG and Research Department of Zoology, Lady Doak College, Madurai, 625 002 Tamilnadu India

## Abstract

Macrofungi belonging to the phylum Basidiomycota are mostly used as medicinal mushrooms in many countries. In the present study, hundred basidiocarp of macrofungi were collected from Tamilnadu during rainy season. The basidiocarp was found in association with root/trunk of living trees, wood log and decayed matter. Among the hundred basidiocarp, 49 were grown into axenic cultures. Notable variations in the macroscopic characteristics of the basidiome and culture morphology were observed. To study the genetic diversity, the molecular taxonomy of the isolates was carried out using internal transcribed spacer (ITS) and 5.8S rRNA gene sequence marker. Thirty-two strains belonging to the order Polyporales, Hymenochataeles and Russuales under the division Basidiomycota were classified based on phylogeny analysis. This study provides first evidence for the occurrence of species *Fulvifomes fastuosus* (LDCMY39 and LDCMY43) and *Ganoderma wiiroense* (LDCMY02, LDCMY08, LDCMY11, LDCMY17 and LDCMY19) from southern India. Molecular evidence for the existence of *Phellinus badius* was given for the first time as well. These data enhance our understanding on the diversity of macrofungi in India, which could be further exploited for biomedical applications.

## Introduction

The kingdom fungi are a distinct group of eukaryotic organisms encompassing about 1.5 M species^1,2^, where 77,000 fungal species are identified by ITS sequence and been reported in GenBank repository^3^. They are identified by filamentous mycelium, absence of motile cells and chlorophyll, presence of chitin-rich cell walls and secretion of external digestive enzymes to degrade the food. Their mode of reproduction is via asexual and sexual spores^4^. These are considered to be the key decomposers of terrestrial ecosystems and known to play crucial ecological role^5–7^. Wild mushrooms from the natural habitat have profound biological and economic impact due to their major role in ecosystem maintenance^8–10^. Destruction of environment is the major threat for fungal diversity; exploration of diversity of macrofungi and their taxonomy are acquired importance for reforestation programmes^11^.

The phylum Basidiomycota includes largely of fleshy fungi (e.g., mushrooms, toadstools, rusts) and ranked second with approximately 23,000 species^4^. Abundant growth of Basidiomycetes are prevalent in the rainy seasons where the environmental conditions such as temperature, relative humidity and sunshine are favourable, which aids them in the breakdown of dead organic tissue^12^. These are the potential indicators of environmental quality^13^. Many fleshy fungi are edible and harmless, but few are poisonous^14^. However, approximately 700 species of Basidiomycetes were reported to exhibit notable pharmacological activities^15,16^. These mainly aids in immune system enhancement, regulation of biorhythm, maintenance of homeostasis and are considered to be the biofactor of effective compounds to cure various diseases as anti-fungal, anti-inflammatory, anti-tumor, anti-viral, anti-bacterial, hepatoprotective, anti-diabetic, hypolipedemic, anti-thrombotic and hypotensive activities^17,18^. Though countless number of macrofungi demonstrates an array of medicinal values only a small fraction has been subjected to scientific examination.

India is rich in fungal biodiversity and consists of one-third of global fungal diversity in which only 50% is characterized and explored^19^. Until 1975, study on mushrooms was neglected in states such as Tamil Nadu, Kerala, Karnataka, and Andhra Pradesh in South India. Natarajan and colleagues^20^ worked on the prospection of mushrooms from southern and south-western region excluding Kerala and, listed 230 agaric and bolete species belonged to 67 genera.

The diversity of Basidiomycetes is studied by classical and molecular methods. It involved collection of basidiome, *in vitro* culture, molecular identification, and preservation of the macrofungi. Classical taxonomy of macrofungi involves description of macro- and micro-morphological characters such as attachment of basidiocarp, types of basidiocarp, pileus surface, margin, pore surface, hyphal system, setae, basidia, basidiospore and reaction to KOH, Meltzer’s reagent etc.^21–23^. Traditional survey alone cannot detect many species of fungi, as they do not produce visible basidicarp or species-specific characteristics. Those can be studied using molecular methods^24–26^. The focus of the present study was to explore the diversity of ethnomycologically important Basidiomycetes in Southern Tamil Nadu, India and we have employed molecular methods for the identification of macrofungi.

Many methods have been used in molecular systematics of macrofungi namely DNA-DNA hybridization; restriction enzyme analysis - RFLP (restriction fragment length polymorphism), rDNA (nuclear ribosomal DNA), mtDNA (mitochondrial DNA); and sequencing analysis – spacers (ITS-internal transcribed spacer), 5S nuclear rRNA, mitochondrial rRNA^27^. The universal primer for fungal phylogenetics comprised of fungal ribosomal operon: large subunit (26S or 28S), small subunit (18S) and the ITS comprising of ITS1 and ITS2 containing the conserved 5.8S^28–30^. The ITS1 and ITS4 primers amplify the highly variable ITS1 and ITS2 sequences surrounding coding sequence of 5.8S and it’s exclusively specific for basidiomycetes^31,32^. This study focussed on sequencing the entire ITS1, 5.8S rRNA and ITS2 for identification of isolated macrofungi. Based on phylogenetic analysis, thirty-two strains belonging to the division Basidiomycota were classified. This study provided additional information to the present knowledge on the data of diversity of fungi in Tamilnadu and also to understand their bioprospects.

## Results

**Figure 1:**
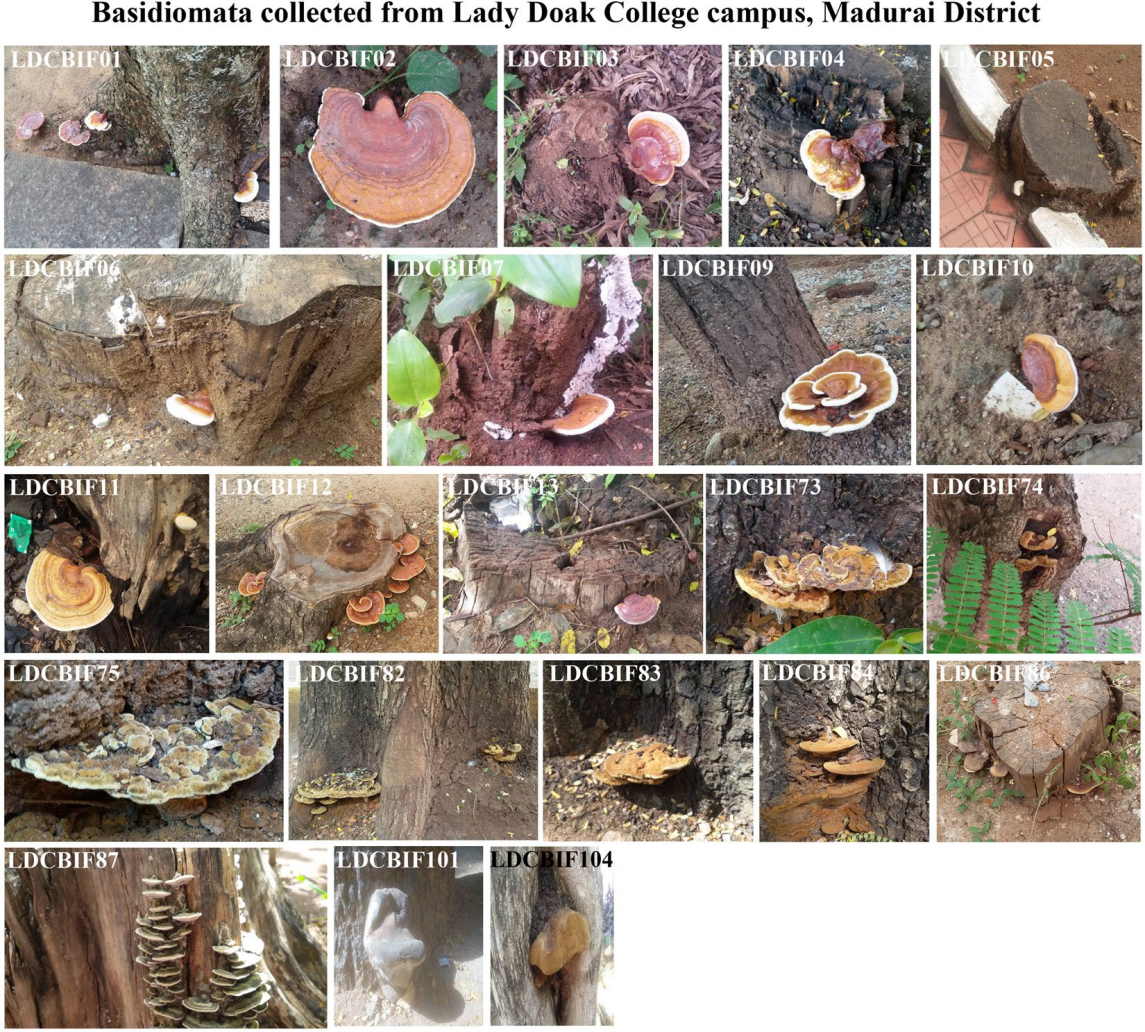
Field photographs of Basidiomata collected from Lady Doak Campus, Madurai District. The macrofungi grown on the host species: *Albizzia* sp., - LDCBIF01, LDCBIF82, LDCBIF83, LDCBIF84; *Azadirachta* sp., - LDCBIF09; *Araccaceae* sp., - LDCBIF101. Few isolates were collected from the decayed matter (LDCBIF02, LDCBIF10 & LDCBIF104) and wood log (LDCBIF03 - LDCBIF07, LDCBIF11 - LDCBIF13, LDCBIF86 & LDCBIF87).

**Figure 2:**
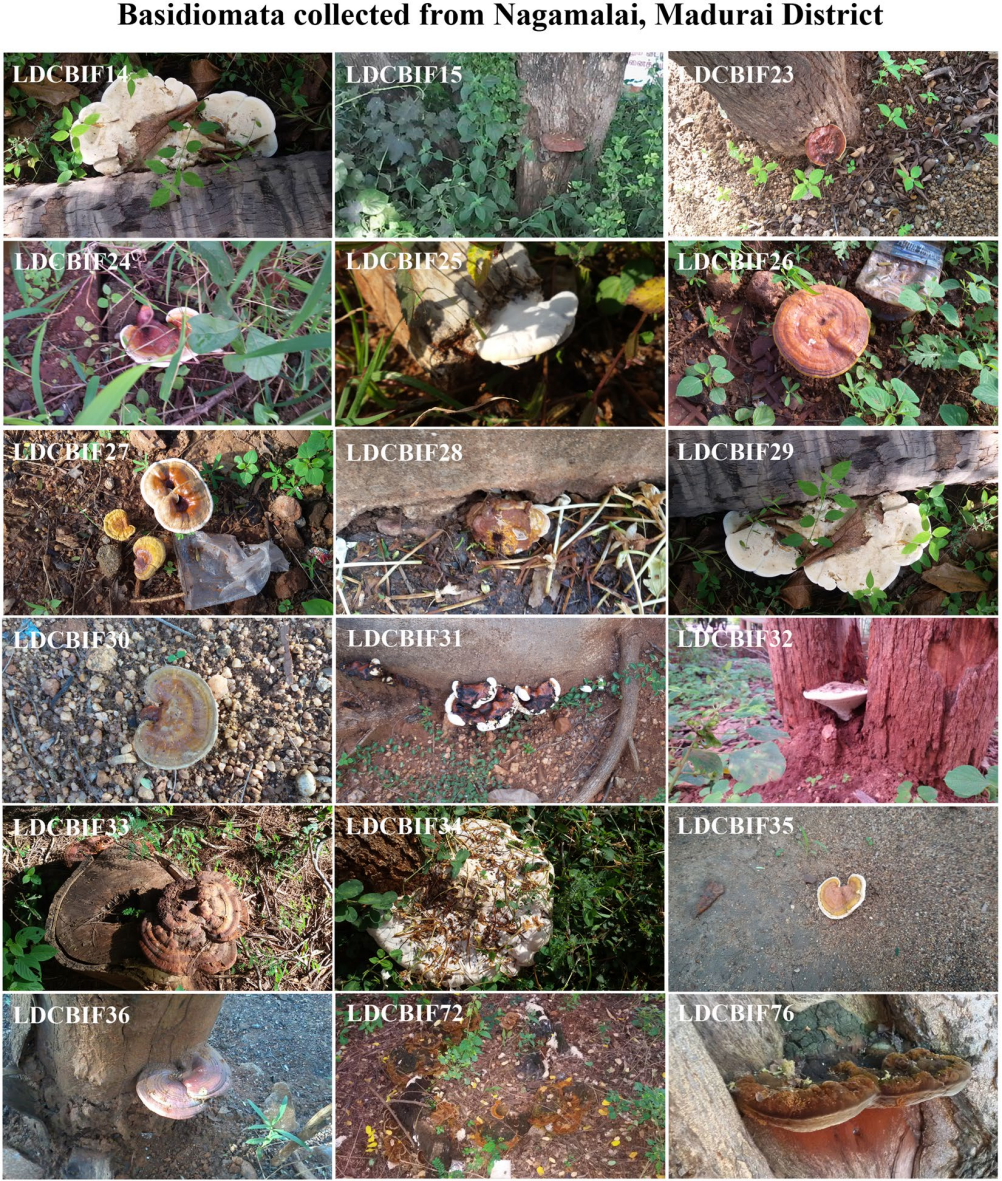
Field photographs of Basidiomata collected from Nagamalai, Madurai District. The macrofungi grown on the host species: *Albizzia* sp., - LDCBIF32, LDCBIF33, LDCBIF35, LDCBIF72, LDCBIF76; *Azadirachta* sp., - LDCBIF30; *Cocos* sp., - LDCBIF24 and *Tamarindus* sp., - LDCBIF15, LDCBIF36. Few isolates were collected from the decayed matter (LDCBIF23, LDCBIF25, LDCBIF26, LDCBIF28, LDCBIF29, LDCBIF31) and wood log (LDCBIF14, LDCBIF27, LDCBIF34).

**Figure 3:**
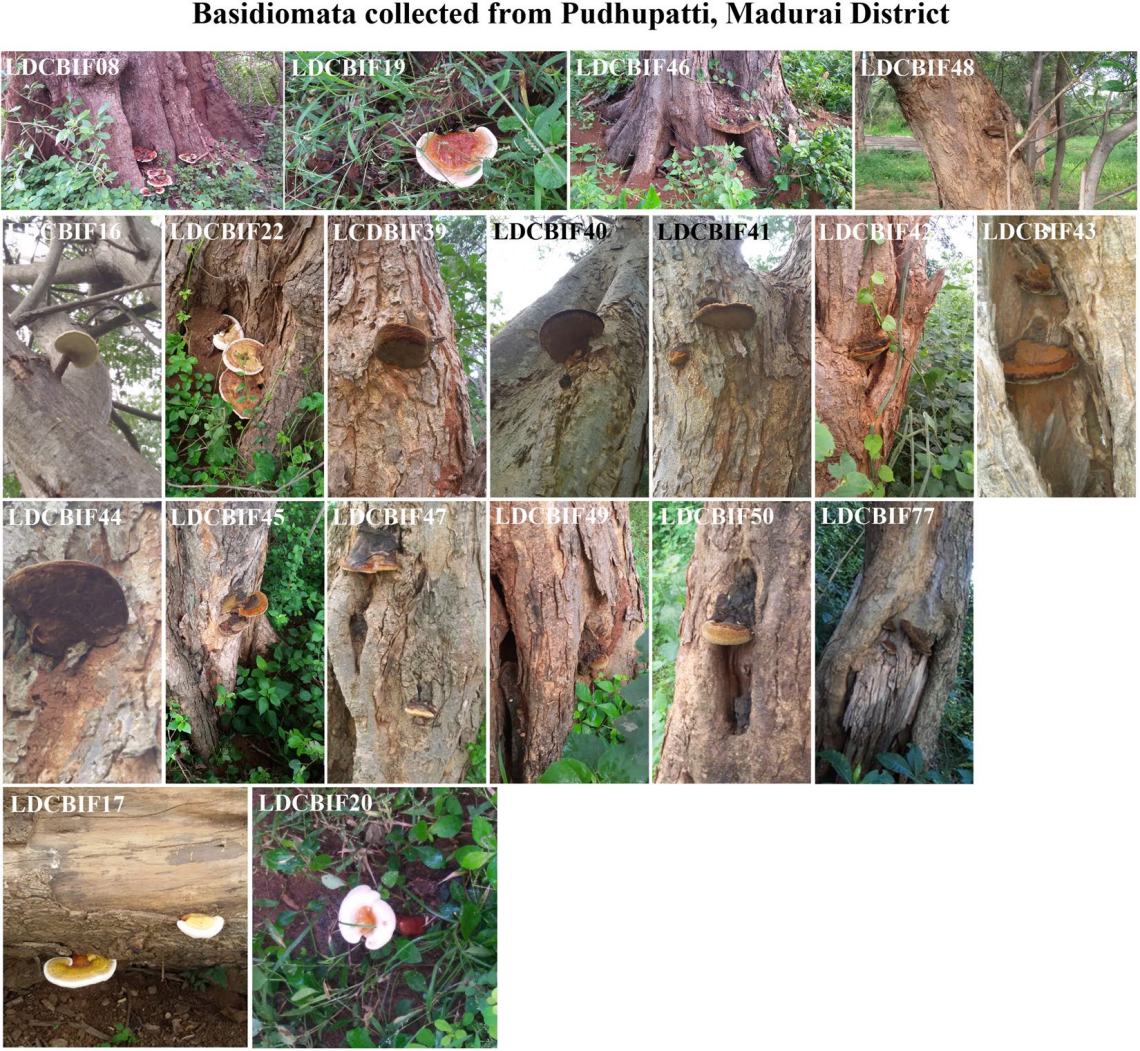
Field photographs of Basidiomata collected from Pudhupatti, Madurai District. The macrofungi grown on the host species: *Albizzia* sp., - LDCBIF16 - LDCBIF22, LDCBIF39, LDCBIF40 - LDCBIF50 and LDCBIF77; *Tamarindus* sp., - LDCBIF08.

**Figure 4:**
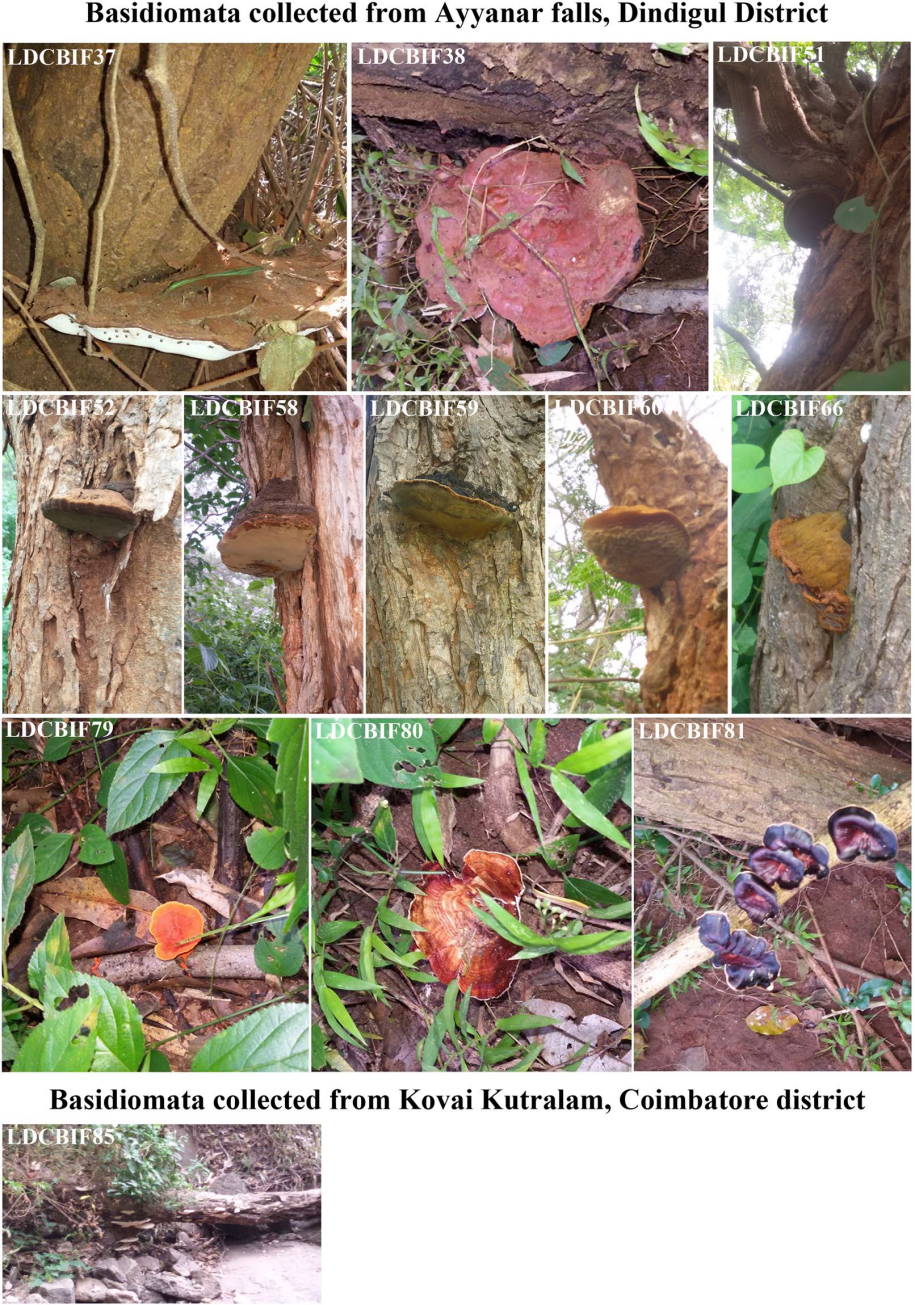
Field photographs of Basidiomata collected from Ayyanar Falls, Dindigul and Kovai kutralam, Coimbatore District. The macrofungi grown on the host species: Ayyanar Falls *- Albizzia* sp., - LDCBIF51, LDCBIF52, LDCBIF58, LDCBIF59, LDCBIF60, LDCBIF66. Few isolates were collected from the decayed matter (LDCBIF79 - LDCBIF81) and wood log (LDCBIF37 & LDCBIF38). Kovai Kutralam - wood log (LDCBIF85)

**Figure 5:**
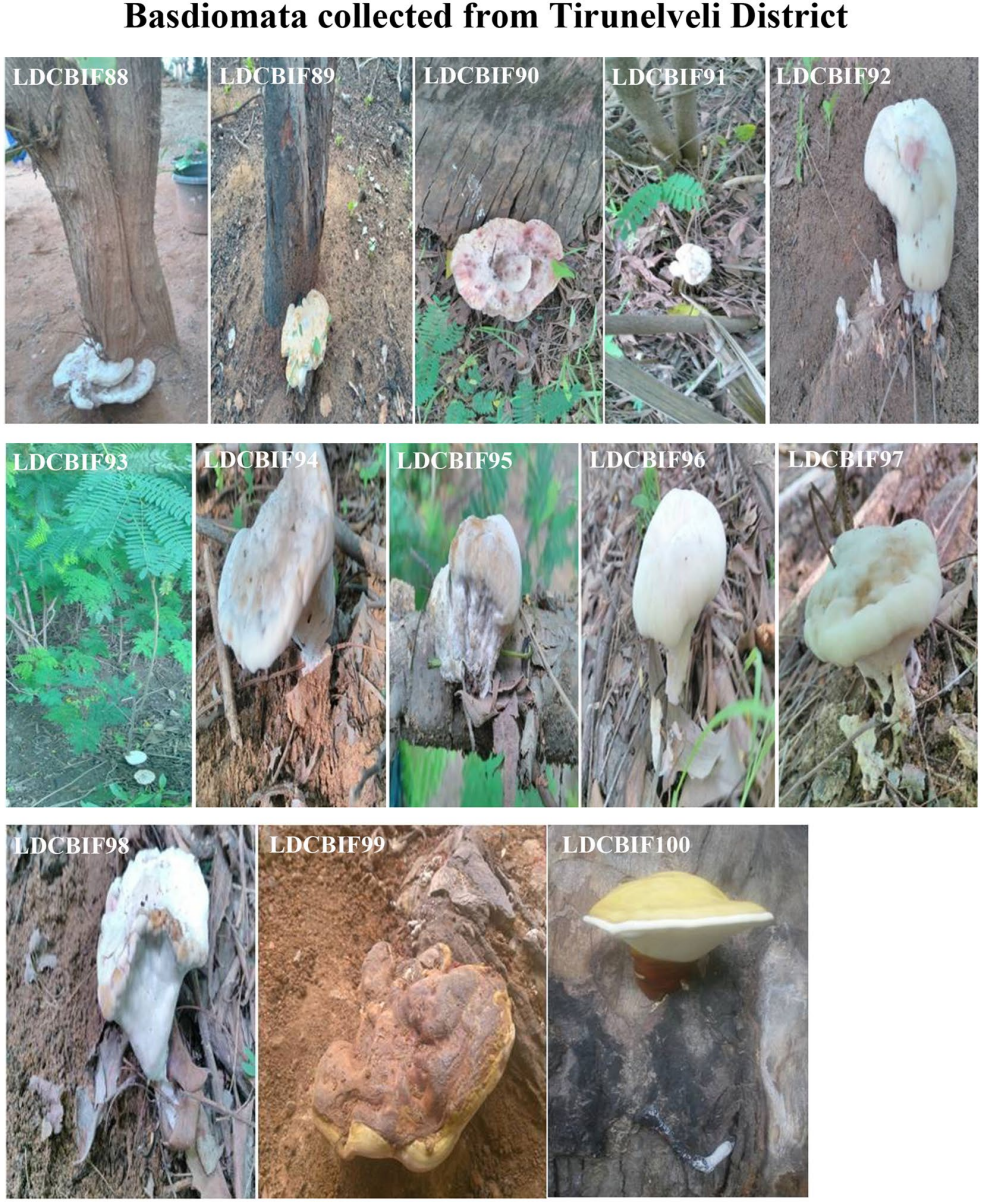
Field photographs of Basidiomata collected from Tirunelveli District. The macrofungi grown on the host species: *Nerium sp*., - LDCBIF88; *Canthium sp*., -LDCBIF89; *Albizzia sp*., - LDCBIF90 - LDCBIF98 and *Tamarindus* sp., - LDCBIF99 & LDCBIF100.

This study is the first report on the occurrence of species *Fulvifomes fastuosus* and *Ganoderma wiiroense* from India. In addition, molecular evidence for the existence of *Phellinus badius* in southern Tamilnadu is also provided. In the present study, hundred basidiomata were collected from different locations: Lady Doak College Campus (Fig. 1), Nagamalai (Fig. 2), Pudhupatti (Fig. 3), Ayyanar falls and Kovai Kutralam (Fig. 4), and Tirunelveli (Fig. 5). The collection details such as habitat, host, attachment pattern and position of basidiome on the tree are mentioned in Table 1. The species richness was found in the following order: Lady Doak College Campus (22%), Pudhupatti (21%), Nagamalai (19%), Ayyanar falls (23%), Tirunelveli (13%), Kovai Kutralam (1%), and Thenkasi (1%). The host of the isolates are as follows: *Albizzia sp*., *Azadirachta sp*., *Canthium dicoccum*, *Cocos nucifera*, *Nerium sp*., *Tamarindus sp*., wood log and decayed leaf litters. In this study, *Albizzia sp*. (58%) was found to be the predominant host. Nearly 56% of the basidiome were associated with tree roots, 36% with tree trunks and 8% with decayed matter. The attachment pattern with the host varied among the isolates: sessile (67%) and stipitate (33%).

**Table 1:** Basidiomata collected.

S. No.	Basidiome Id	Host	Attachment to the Host	Position of basidiome on the tree	Size L * W (in cm)	Xanthochroic
1.	LDCBIF01*	*Albizzia* sp	Stipitate	Root	15, 10.5	−
2.	LDCBIF02*	Decayed material	Stipitate	—	10.5, 7.5	−
3.	LDCBIF03*	Wood Log	Sessile	Root	11, 9	−
4.	LDCBIF04*	Wood Log	Sessile	Root	7.5, 4.5	−
5.	LDCBIF05*	Wood Log	Sessile	Root	NA	−
6.	LDCBIF06*	Wood Log	Sessile	Root	NA	−
7.	LDCBIF07*	Wood Log	Sessile	Root	6, 5	−
8.	LDCBIF08*	*Tamarindus* sp.	Stipitate	Root	NA	−
9.	LDCBIF09*	*Azadirachta* sp.	Stipitate	Root	16, 13.5	−
10.	LDCBIF10*	Decayed material	Sessile	—	4, 5	−
11.	LDCBIF11*	Wood Log	Stipitate	Root	12, 5	−
12.	LDCBIF12*	Wood Log	Stipitate	Root	15, 7	−
13.	LDCBIF13*	Wood Log	Stipitate	Root	9.5, 7	−
14.	LDCBIF14^#^	Wood Log	Sessile	Root	15.2, 8	−
15.	LDCBIF15^#^	*Tamarindus* sp.	Sessile	Root	19, 10.5	−
16.	LDCBIF16^@^	*Albizzia* sp.	Stipitate	Root	4, 2	−
17.	LDCBIF17^@^	*Albizzia* sp.	Stipitate	Root	9, 5	−
18.	LDCBIF18^@^	*Albizzia* sp.	Stipitate	Root	7, 4.5	−
19.	LDCBIF19^@^	*Albizzia* sp.	Stipitate	Root	9, 7	−
20.	LDCBIF20^@^	*Albizzia* sp.	Stipitate	Root	5.5, 3	−
21.	LDCBIF21^@^	*Albizzia* sp.	Stipitate	Root	5,3	−
22.	LDCBIF22^@^	*Albizzia* sp.	Sessile	Root	12,7.5	−
23.	LDCBIF23^#^	Decayed material	Stipitate	Root	7.5, 6	−
24.	LDCBIF24^#^	*Cocos sp*.	Sessile	Root	39, 20	−
25.	LDCBIF25^#^	Decayed material	Stipitate	Root	6, 3	−
26.	LDCBIF26^#^	Decayed material	Stipitate	Root	7.8, 6	−
27.	LDCBIF27^#^	Wood Log	Sessile	Root	NA	−
28.	LDCBIF28^#^	Decayed material	Stipitate	Root	8.5, 7	−
29.	LDCBIF29^#^	Decayed material	Stipitate	Root	7,6	−
30.	LDCBIF30^#^	*Azadirachta sp*.	Stipitate	Root	5.8, 3.5	−
31.	LDCBIF31^#^	Decayed material	Sessile	Root	5, 3.5	−
32.	LDCBIF32^#^	*Albizzia sp*.	Sessile	Root	25, 16.5	−
33.	LDCBIF33^#^	*Albizzia sp*.	sessile	Root	11.5, 7	−
34.	LDCBIF34^#^	Wood Log	Sessile	Root	4.5, 2.5	−
35.	LDCBIF35^#^	*Albizzia sp*.	Sessile	Root	14, 6	−
36.	LDCBIF36^#^	*Tamarindus sp*.	Sessile	Trunk	15.5, 10	−
37.	LDCBIF37^$^	Wood Log	Sessile	Root	25, 18	−
38.	LDCBIF38^$^	Wood Log	Sessile	Root	12, 10.5	−
39.	LDCBIF39^@^	*Albizzia* sp.	Sessile	Trunk	4.5, 3	+
40.	LDCBIF40^@^	*Albizzia* sp.	Sessile	Trunk	4.8, 2.8	+
41.	LDCBIF41^@^	*Albizzia* sp.	Sessile	Trunk	4.5, 3.5	+
42.	LDCBIF42^@^	*Albizzia* sp.	Sessile	Trunk	5.5, 4	+
43.	LDCBIF43^@^	*Albizzia* sp.	Sessile	Trunk	5, 3.5	+
44.	LDCBIF44^@^	*Albizzia* sp.	Sessile	Trunk	10, 6	+
45.	LDCBIF45^@^	*Albizzia* sp.	Sessile	Trunk	7, 3.5	+
46.	LDCBIF46^@^	*Albizzia* sp.	Sessile	Trunk	6, 4.5	+
47.	LDCBIF47^@^	*Albizzia* sp.	Sessile	Trunk	12, 5.6	+
48.	LDCBIF48^@^	*Albizzia* sp.	Sessile	Trunk	15, 6.5	+
49.	LDCBIF49^@^	*Albizzia* sp.	Sessile	Trunk	19.5, 9	+
50.	LDCBIF50^@^	*Albizzia* sp.	Sessile	Trunk	10.5, 6	+
51.	LDCBIF51^$^	*Albizzia* sp.	Sessile	Trunk	12, 9.5	+
52.	LDCBIF52^$^	*Albizzia* sp.	Sessile	Trunk	10, 5.5	+
53.	LDCBIF53^$^	*Albizzia* sp.	Sessile	Trunk	14, 8	+
54.	LDCBIF54^$^	*Albizzia* sp.	Sessile	Trunk	11.5,7	+
55.	LDCBIF55^$^	*Albizzia* sp.	Sessile	Trunk	5.5, 4.5	+
56.	LDCBIF56^$^	*Albizzia* sp.	Sessile	Trunk	7, 4	+
57.	LDCBIF57^$^	*Albizzia* sp.	Sessile	Trunk	9.5, 6	+
58.	LDCBIF58^$^	*Albizzia* sp.	Sessile	Trunk	13, 5.5	+
59.	LDCBIF59^$^	*Albizzia* sp.	Sessile	Trunk	9, 6	+
60.	LDCBIF60^$^	*Albizzia* sp.	Sessile	Trunk	6, 4.5	+
61.	LDCBIF61^$^	*Albizzia* sp.	Sessile	Trunk	4, 2	+
62.	LDCBIF62^$^	*Albizzia* sp.	Sessile	Trunk	7.5, 4	+
63.	LDCBIF63^$^	*Albizzia* sp.	Sessile	Trunk	5, 2.5	+
64.	LDCBIF64^$^	*Albizzia* sp.	Sessile	Trunk	6.5, 3	+
65.	LDCBIF65^$^	*Albizzia* sp.	Sessile	Trunk	7, 5	+
66.	LDCBIF66^$^	*Albizzia* sp.	Sessile	Trunk	5, 5	+
67.	LDCBIF67^$^	*Albizzia* sp.	Sessile	Trunk	11, 7	+
68.	LDCBIF68^$^	*Albizzia* sp.	Sessile	Trunk	11, 4.8	+
69.	LDCBIF71^#^	*Albizzia* sp.	Sessile	Trunk	8.5, 5	+
70.	LDCBIF72^#^	*Albizzia* sp.	Sessile	Trunk	5.5, 3.5	+
71.	LDCBIF73*	*Albizzia* sp.	Sessile	Root	6, 5	+
72.	LDCBIF74*	*Albizzia* sp.	Sessile	Trunk	3, 2	+
73.	LDCBIF75*	*Albizzia* sp.	Sessile	Root	5.5, 3	+
74.	LDCBIF76^#^	*Albizzia* sp.	Sessile	Trunk	6, 4.5	+
75.	LDCBIF77^@^	*Albizzia* sp.	Sessile	Trunk	NA	+
76.	LDCBIF78^≠^	Wood Log	Sessile	—	11.5, 7	−
77.	LDCBIF79^#^	Decayed material	Stipitate	—	3, 3	−
78.	LDCBIF80^#^	Decayed material	Stipitate	—	7, 5	−
79.	LDCBIF81^#^	Decayed material	Stipitate	—	6, 5.8	−
80.	LDCBIF82*	*Albizzia a* sp.	Sessile	Root	4, 2.5	+
81.	LDCBIF83*	*Albizzia* sp.	Sessile	Root	NA	+
82.	LDCBIF84*	*Albizzia* sp.	Sessile	Root	NA	+
83.	LDCBIF85^€^	Wood Log	Sessile	—	NA	−
84.	LDCBIF86*	Wood Log	Stipitate	Root	NA	−
85.	LDCBIF87*	Wood Log	Sessile	Trunk	NA	−
86.	LDCBIF88^®^	*Nerium sp*.	Sessile	Root	16, 8.4	−
87.	LDCBIF89^®^	*Canthium sp*.	Sessile	Root	11, 8	−
88.	LDCBIF90^®^	*Cocos sp*.	Stipitate	Root	9.1, 8	−
89.	LDCBIF91^®^	*Cocos sp*.	Stipitate	Root	3, 3.5	−
90.	LDCBIF92^®^	*Albizzia sp*.	Stipitate	Root	8, 5	−
91.	LDCBIF93^®^	*Albizzia sp*.	Stipitate	Root	5, 4.3	−
92.	LDCBIF94^®^	*Albizzia sp*.	Stipitate	Root	7.2, 5.1	−
93.	LDCBIF95^®^	*Albizzia sp*.	Sessile	Root	3, 2.8	−
94.	LDCBIF96^®^	*Albizzia sp*.	Stipitate	Root	4, 3.8	−
95.	LDCBIF97^®^	*Albizzia sp*.	Stipitate	Root	7, 5.2	−
96.	LDCBIF98^®^	*Albizzia sp*.	Stipitate	Root	4.8, 3.4	−
97.	LDCBIF99^®^	*Tamarindus* sp.	Sessile	Root	10.8, 6	−
98.	LDCBIF100^®^	*Tamarindus* sp.	Stipitate	Root	4.4, 4	−
99.	LDCBIF101*	*Araccaceae* sp.	Sessile	Root	7.8, 6.8	−
100.	LDCBIF104*	Decayed Material	Stipitate	—	NA	−

^$, €,^ *^, #, ≠, @, ®^used to denote the strains collected from different places. ^$^Ayyanar falls; ^€^Coimbatore; *Lady Doak College Campus; ^#^Nagamalai; ^≠^Thenkasi; ^@^Pudhupatti; ^®^Tirunelveli.

**Figure 6:**
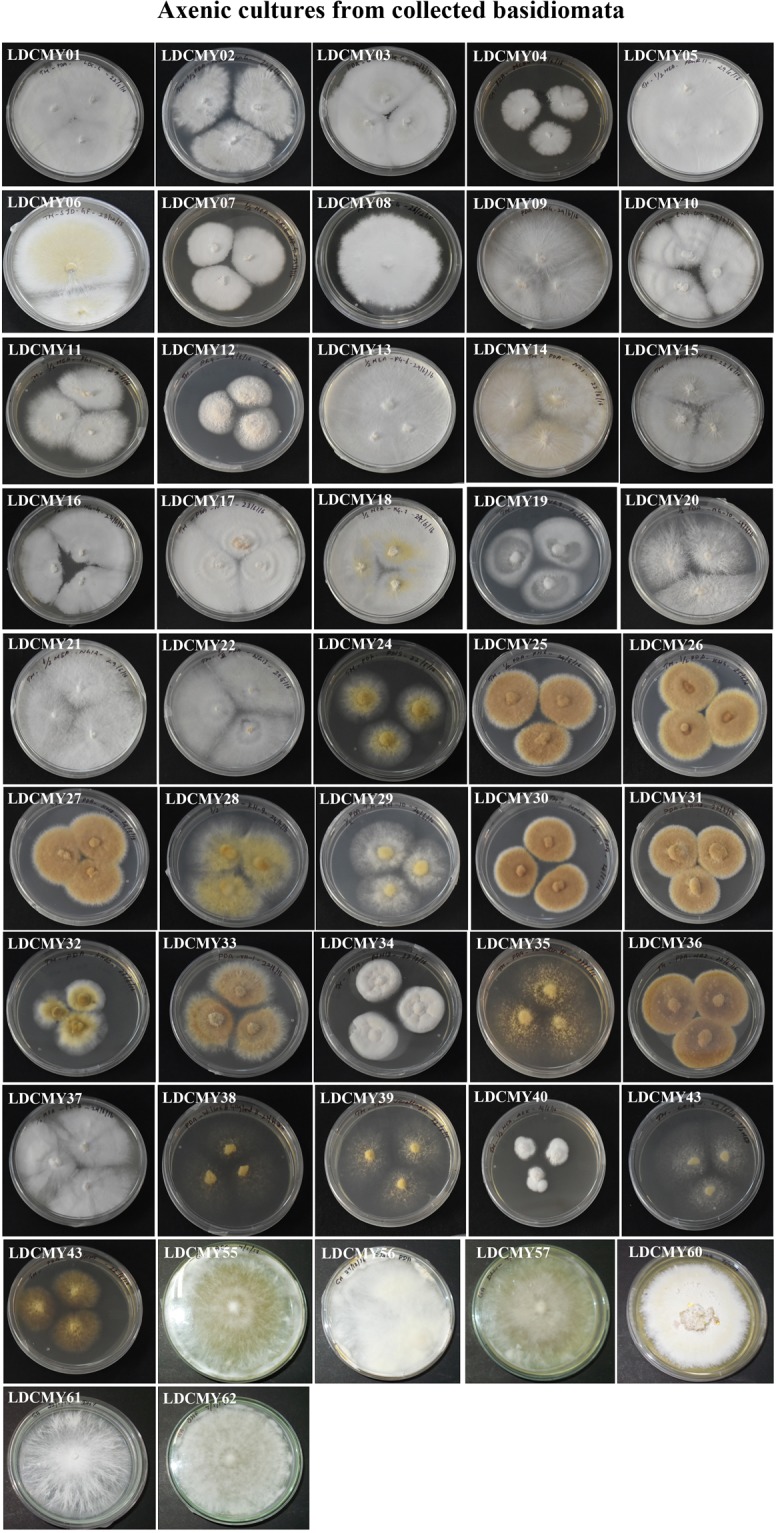
Axenic culture of collected basidiomata. The mycelium culture on PDA plates. Variations in growth and the color of the mycelium was observed (See Table 2). The identified strains by sequencing; *Amylosporous sp*. *-* LDCMY57 & LDCMY58; *Coriolopsis caperata -* LDCMY42; *Fomitopsis ostreiformis -* LDCMY21; *Fulvifomes fastuosus -* LDCMY39, LDCMY43; *Ganoderma resinaceum -* LDCMY01; *Ganoderma sp*. *-* LDCMY04, LDCMY05, LDCMY06; LDCMY12, LDCMY14, LDCMY16, LDCMY18, LDCMY22, LDCMY41. *Ganoderma wiiroense -* LDCMY19, LDCMY08, LDCMY11, LDCMY17 and LDCMY02; *Inonotus rickii -* LDCMY52; *Phellinus badius -* LDCMY36; *Phellinus sp*. *-* LDCMY23, LDCMY24, LDCMY27, LDCMY28, LDCMY29, LDCMY31, LDCMY34, LDCMY45; *Trametes elegans -* LDCMY37.

Among the hundred basidiome collected only forty-nine isolates (49%) could be grown in axenic cultures. The mycelial growth significantly varied from 7 days to 30 days. The colour of the mycelia varies for each strain: white, orange white, yellowish white, pale yellow, greyish orange, light yellow, pale orange and brownish orange (Fig. 6, Table 2). The pure cultures of all isolates were stored in mineral oil till further use.

**Table 2:** Growth and characteristics of mycelium culture.

S. No.	Basidiome Id	Strain Id	Mycelial growth in PDA plates			
			Initial radial expansion (in mm)	Complete colonization (in days)	Front Color	Reverse Color
1.	LDCBIF01	LDCMY01*	**23**.**44 ± 0**.**24**	7	White	Pale Yellow
2.	LDCBIF02	LDCMY02*	**22**.**00 ± 0**.**15**	7	Orange White	Pale Yellow
3.	LDCBIF03	LDCMY03*	**37**.**66 ± 0**.**20**	7	White	Pale Yellow
4.	LDCBIF04	LDCMY04*	**18**.**00 ± 0**.**26**	7	White	White
5.	LDCBIF06	LDCMY41*	**25**.**33 ± 0**.**15**	7	White	Pale Yellow
6.	LDCBIF08	LDCMY05*	**19**.**33 ± 0**.**05**	7	Orange White	Pale Yellow
7.	LDCBIF09	LDCMY06*	**25**.**00 ± 0**.**15**	7	White	Pale Yellow
8.	LDCBIF10	LDCMY07*	**22**.**33 ± 0**.**25**	7	White	Light Yellow
9.	LDCBIF11	LDCMY08*	**22**.**00 ± 0**.**55**	7	White	Light Yellow
10.	LDCBIF12	LDCMY09*	**36**.**66 ± 0**.**55**	7	White	Pale Yellow
11.	LDCBIF13	LDCMY10*	**32**.**66 ± 0**.**15**	7	White	Light Yellow
12.	LDCBIF16	LDCMY11^@^	**25**.**66 ± 0**.**15**	7	White	Light Yellow
13.	LDCBIF19	LDCMY12^@^	**21**.**33 ± 0**.**11**	7	White	Light Yellow
14.	LDCBIF21	LDCMY13^@^	**41**.**33 ± 0**.**92**	7	White	Pale Yellow
15.	LDCBIF23	LDCMY14^#^	**29**.**66 ± 0**.**20**	7	White	Light Orange
16.	LDCBIF25	LDCMY15^#^	**24**.**00 ± 0**.**30**	7	White	Pale Yellow
17.	LDCBIF26	LDCMY16^#^	**19**.**33 ± 0**.**23**	7	Yellowish White	Greyish Yellow
18.	LDCBIF28	LDCMY17^#^	**23**.**00 ± 0**.**26**	7	White	Pale Yellow
19.	LDCBIF29	LDCMY18^#^	**32**.**00 ± 0**.**32**	7	Pale Yellow	Light Orange
20.	LDCBIF31	LDCMY19^#^	**37**.**33 ± 0**.**25**	7	White	White
21.	LDCBIF32	LDCMY20^#^	**34**.**00 ± 0**.**36**	7	White	Pale Yellow
22.	LDCBIF34	LDCMY21^#^	**33**.**00 ± 0**.**75**	7	White	Pale Yellow
23.	LDCBIF35	LDCMY22^#^	**28**.**33 ± 0**.**05**	7	White	White
24.	LDCBIF39	LDCMY23^@^	18.00 ± 0.10	30	Greyish Orange	Greyish Orange
25.	LDCBIF43	LDCMY24^@^	18.00 ± 0.15	27	Light Yellow	Greyish Yellow
26.	LDCBIF44	LDCMY25^@^	23.00 ± 0.20	14	Light Yellow	Greyish Yellow
27.	LDCBIF55	LDCMY26^$^	26.66 ± 0.05	17	Greyish Orange	Greyish Orange
28.	LDCBIF58	LDCMY27^$^	22.66 ± 0.11	17	Greyish Orange	Greyish Orange
29.	LDCBIF59	LDCMY28^$^	27.00 ± 0.51	17	Greyish Orange	Greyish Orange
30.	LDCBIF60	LDCMY29^$^	22.33 ± 0.47	17	Light Yellow	Light Yellow
31.	LDCBIF62	LDCMY30^$^	22.66 ± 0.32	17	Greyish Orange	Greyish Orange
32.	LDCBIF66	LDCMY31^$^	22.00 ± 0.17	30	Pale Orange	Light Orange
33.	LDCBIF68	LDCMY32^$^	34.00 ± 0.45	27	Light Yellow	Greyish Yellow
34.	LDCBIF71	LDCMY44^#^	27.33 ± 0.15	20	Brownish Orange	Deep Orange
35.	LDCBIF72	LDCMY34^#^	21.66 ± 0.25	19	Light Yellow	Brownish Yellow
36.	LDCBIF73	LDCMY35*	37.66 ± 0.40	17	Greyish Orange	Greyish Orange
37.	LDCBIF74	LDCMY43*	28.00 ± 0.10	20	Brownish Orange	Deep Orange
38.	LDCBIF77	LDCMY36^@^	23.33 ± 0.05	15	Greyish Orange	Greyish Orange
39.	LDCBIF78	LDCMY37^≠^	1**7**.**00 ± 0**.**26**	5	White	Pale Yellow
40.	LDCBIF82	LDCMY38*	27.33 ± 0.45	17	Greyish Yellow	Deep Orange
41.	LDCBIF84	LDCMY39*	25.33 ± 0.20	20	Brownish Orange	Deep Orange
42.	LDCBIF85	LDCMY40*	**18**.**00 ± 0**.**43**	5	White	Light Yellow
43.	LDCBIF86	LDCMY41*	**21**.**00 ± 0**.**39**	7	White	Pale Yellow
44.	LDCBIF87	LDCMY42^£^	**32**.**00 ± 0**.**21**	5	White	Light Yellow
45.	LDCBIF88	LDCMY57^®^	**27**.**00 ± 0**.**10**	5	White	Pale Yellow
46.	LDCBIF96	LDCMY58^®^	**17**.**33 ± 0**.**15**	5	White	Pale Yellow
47.	LDCBIF100	LDCMY60^®^	**18**.**00 ± 0**.**43**	7	White	Pale Yellow
48.	LDCBIF101	LDCMY61^®^	**26**.**66 ± 0**.**25**	7	White	Pale Yellow
49.	LDCBIF104	LDCMY62*	**21**.**66 ± 0**.**11**	7	White	Pale Yellow

^**$**,€,*,**#**,≠,**@**,**®**^**U**sed to denote the strains collected from different places. ^$^Ayyanar falls; ^€^Coimbatore; *Lady Doak College Campus; ^#^Nagamalai; ^≠^Thenkasi; ^@^Pudhupatti; ^®^Tirunelveli.

The radial expansion was measured on the 3^rd^ day (shown in bold) and 7^th^ day. The measurements are given in mean ± SD. The total number of days taken for complete colonization (80 mm) in PDA medium varied among the isolates and ranged from 5–30 days for different strains.

**Figure 7:**
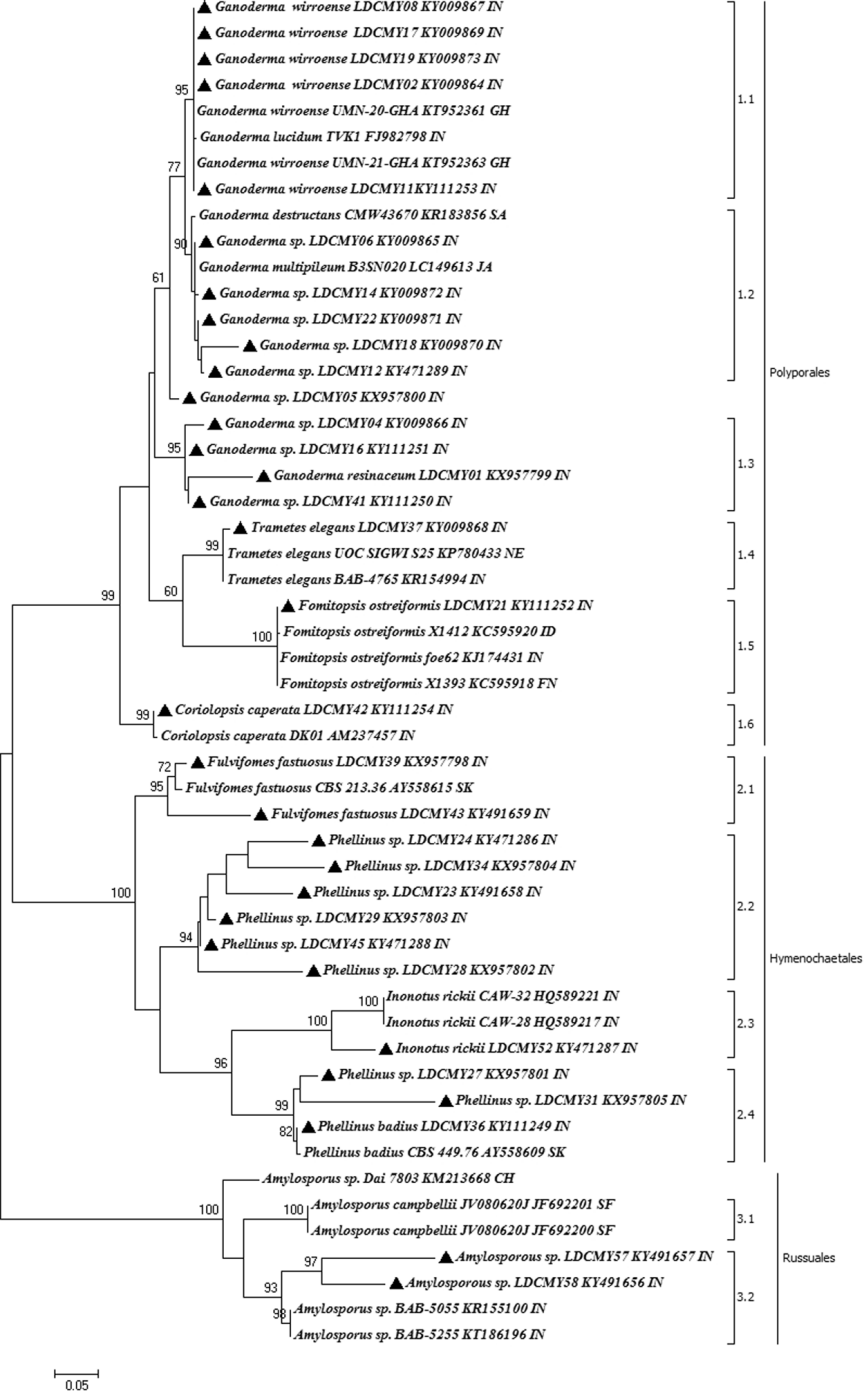
The evolutionary relationship was inferred using the maximum Likelihood method in MEGA6. The analysis involved 52 nucleotide sequences; thirty two sequences generated in this study are highlighted. The initial trees were obtained with the random addition of sequences. All positions containing gaps and missing data were eliminated. Numerical values above the internodes are the percentage of 1000 bootstrap replications. Bootstrap values higher than 60% are indicated. Scale bar 0.05 represents nucleotide substitutions per position. Three clades were predicted Clade 1: Polyporales; Clade 2: Hymenochaetales; Clade 3: Russuales. The abbreviated letters next to accession number indicates the localities from which the sample is collected: IN - India, GH - Ghana, CH- China, ID - Indonesia, FL - Finland, NE - Nepal, SA - South Africa, SF - South Florida, SK - South Korea, SL - Sri Lanka. The diversity within subpopulation was predicted as 0.1, the diversity within entire population - 0.3 with a Mean inter population Diversity - 0.3 and Coefficient of differentiation - 0.8.

Genomic DNA was obtained and 5.8S ribosomal RNA gene segment was amplified using sequence specific primers. Thirty-two isolates were successfully sequenced and the size of the amplicon ranged from 599 bp to 902 bp. The sequences were deposited in GenBank and accession numbers were obtained (Table 3). Variation in genetic makeup was observed among the isolates from the same environment. Molecular phylogentic analysis was carried out using 52 ITS sequences in which 20 reference sequences were retrieved from GenBank, NCBI to clarify the variation among the sequences. The phylogenetic tree constructed using maximum likelihood (ML) method (Fig. 7). The basidiomycete species were clustered into three clades: Clade 1 - Polyporales, Clade 2 - Hymenochaetales and Clade 3 - Russuales. The three clades are detailed below:

**Table 3:** Species and their GenBank accession number used for constructing molecular phylogeny.

S.No	Organism Name	Strain/Isolate Name	Source of DNA	Geographical Origin	Sequence Length (ITS1/ITS4)	Accession No
1.	*Amylosporous sp*.	LDCMY58^®^	Mycelium	Tirunelveli, South India	741	KY491656
2.	*Amylosporous sp*.	LDCMY57^®^	Mycelium	Tirunelveli, South India	774	KY491657
3.	*Amylosporus sp*.	BAB-5055	—	India	897	KR155100
4.	*Amylosporus sp*.	BAB-5255	—	India	775	KT186196
5.	Amylosporus sp.	Dai 7803	—	China	748	KM213668
6.	Amylosporus campbellii	JV080620J	—	Southern Florida	807	JF692201
7.	Amylosporus campbellii	JV080620J	—	Southern Florida	810	JF692200
8.	*Coriolopsis caperata*	LDCMY42*	Mycelium	Lady Doak College Campus, Madurai, South India	614	KY111254
9.	Coriolopsis caperata	DK01	—	New Delhi	585	AM237457
10.	*Fomitopsis ostreiformis*	LDCMY21^#^	Mycelium	Nagamalai, Madurai, South India	599	KY111252
11.	Fomitopsis ostreiformis	X1412	—	Indonesia	1600	KC595920
12.	*Fomitopsis ostreiformis*	foe62	—	Karnataka- India	636	KJ174431
13.	*Fomitopsis ostreiformis*	X1393	—	Finland	1600	*KC595918*
14.	*Fulvifomes fastuosus*	LDCMY39*	Mycelium	Lady Doak College Campus, Madurai, South India	756	KX957798
15.	*Fulvifomes fastuosus*	LDCMY43*	Mycelium	Lady Doak College Campus, Madurai, South India	738	KY491659
16.	*Fulvifomes fastuosus*	CBS 213.36	—	South Korea	768	AY558615
17.	*Ganoderma destructans*	CMW43670	—	South Africa	640	KR183856
18.	*Ganoderma lucidum*	TVK1	—	India	603	FJ982798
19.	*Ganoderma multipileum*	B3SN020	—	Japan	832	LC149613
20.	*Ganoderma resinaceum*	LDCMY01*	Mycelium	Lady Doak College Campus, Madurai, South India	614	KX957799
21.	*Ganoderma sp*.	LDCMY04*	Mycelium	Lady Doak College Campus, Madurai, South India	610	KY009866
22.	*Ganoderma sp*.	LDCMY05*	Mycelium	Lady Doak College Campus, Madurai, South India	620	KX957800
23.	*Ganoderma sp*.	LDCMY06*	Mycelium	Lady Doak College Campus, Madurai, South India	608	KY009865
24.	*Ganoderma sp*.	LDCMY12^@^	Mycelium	Pudhupatti, South India	606	KY471289
25.	*Ganoderma sp*.	LDCMY16^#^	Mycelium	Nagamalai, Madurai, South India	607	KY111251
26.	*Ganoderma sp*.	LDCMY18^#^	Mycelium	Nagamalai, Madurai, South India	722	KY009870
27.	*Ganoderma sp*.	LDCMY22^#^	Mycelium	Nagamalai, Madurai, South India	619	KY009871
28.	*Ganoderma sp*.	LDCMY14^#^	Mycelium	Nagamalai, Madurai, South India	614	KY009872
29.	*Ganoderma sp*.	LDCMY41*	Mycelium	Lady Doak College Campus, Madurai, South India	642	KY111250
30.	*Ganoderma wiiroense*	LDCMY02*	Mycelium	Lady Doak College Campus, Madurai, South India	608	KY009864
31.	*Ganoderma wiiroense*	LDCMY08*	Mycelium	Lady Doak College Campus, Madurai, South India	618	KY009867
32.	*Ganoderma wiiroense*	LDCMY11^@^	Mycelium	Pudhupatti, South India	611	KY111253
33.	*Ganoderma wiiroense*	LDCMY17^#^	Mycelium	Nagamalai, Madurai, South India	612	KY009869
34.	*Ganoderma wiiroense*	LDCMY19^#^	Mycelium	Nagamalai, Madurai, South India	647	KY009873
35.	*Ganoderma wiiroense*	UMN-20-GHA	—	USA	769	KT952361
36.	*Ganoderma wiiroense*	UMN-21-GHA	—	USA	722	KT952363
37.	*Inonotus rickii*	LDCMY52^$^	Basidiome	Ayyanar falls, Dindigul, South India	902	KY471287
38.	Inonotus rickii	CAW-32	—	Rajasthan- India	747	HQ589221
39.	Inonotus rickii	CAW-28	—	Rajasthan - India	750	HQ589217
40.	*Phellinus badius*	LDCMY36^@^	Mycelium	Pudhupatti, South India	688	KY111249
41.	*Phellinus badius*	CBS 449.76	—	South Korea	714	AY558609
42.	*Phellinus sp*.	LDCMY23^@^	Mycelium	Pudhupatti, South India	709	KY491658
43.	*Phellinus sp*.	LDCMY 24^@^	Mycelium	Pudhupatti, South India	668	KY471286
44.	*Phellinus sp*.	LDCMY27^$^	Mycelium	Ayyanar falls, Dindigul, South India	662	KX957801
45.	*Phellinus sp*.	LDCMY28^$^	Mycelium	Ayyanar falls, Dindigul, South India	693	KX957802
46.	*Phellinus sp*.	LDCMY29^$^	Mycelium	Ayyanar falls, Dindigul, South India	683	KX957803
47.	*Phellinus sp*.	LDCMY31^$^	Mycelium	Ayyanar falls, Dindigul, South India	685	KX957805
48.	*Phellinus sp*.	LDCMY34^#^	Mycelium	Nagamalai, Madurai, South India	681	KX957804
49.	*Phellinus sp*.	LDCMY45^$^	Basidiome	Ayyanar falls, Dindigul, South India	677	KY471288
50.	*Trametes elegans*	LDCMY37^≠^	Mycelium	Thenkasi, South India	606	KY009868
51.	Trametes elegans	UOC SIGWI S25	—	Nepal	655	KP780433
52.	Trametes elegans	BAB-4765	—	India	637	KR154994

^$,€,*,#,≠,@,®^Used to denote the sequence data generated from the strains collected from different places. ^$^Ayyanar falls; ^€^Kovai kutralam *Lady Doak College Campus; ^#^Nagamalai; ^≠^Thenkasi; ^@^Pudhupatti; ®Tirunelveli.

Clade 1: Polyporales - Found in all study sites except Ayyanar falls. Eighteen strains were grouped under this clade and fifteen sequences were further categorised under the family Ganodermataceae, two under Polyporaceae and one in Fomitopsidaceae. The isolated strains belong to the Polyporales were *Coriolopsis caperata*, *Fomitopsis ostreiformis*, *Ganoderma resinaceum*, *Ganoderma sp*., *Ganoderma wiiroense* and *Trametes elegans*. *Coriolopsis caperata LDCMY42* collected from Nagamalai showed 99% similarity with the strain *Coriolopsis caperata DK01* (AM237457). Monophyletic origin of *Fomitopsis ostreiformis* was determined with 100% bootstrap support. Five strains were identified as *Ganoderma wiiroense* (LDCMY19, LDCMY08, LDCMY11, LDCMY17 and LDCMY02) and showed highest similarity with the strains reported from United States of America (KT952361 and KT952363). Variations in the genetic makeup as well in the morphology of the *Ganoderma wiiroense* strains were observed. Majority of the *Ganoderma* strains were found to be stipitate. Based on molecular analysis, this is the first evidence for the occurrence of *Ganoderma wiiroense* from India.

The Clade 1 was supported by 99% bootstrap value and it was further categorized into 6 groups (1.1–1.6). Three groups (1.1–1.3) in this clade consisted of strains from *Ganoderma sp*. Five strains of *Ganoderma wiiroense* were grouped in 1.1 and supported by 95% bootstrap value. The mean difference between the sequences in this group was very low (0.000878851). The group 1.2 included *Ganoderma sp*., which is supported by 90% bootstrap with the mean difference of 0.019876893. The group 1.3 included *Ganoderma sp*. from different places, which was supported by 95% bootstrap value with the mean difference of 0.049142826. The group 1.4 included *Trametes elegans* LDCMY37, Thenkasi showed similarity with two strains reported from Nepal and India, and supported by 99% bootstrap value with the mean difference of 0.004707472. The group 1.5 included *Fomitopsis ostreiformis* LDCMY21 isolated from Nagamalai supported by 100% bootstrap value with the mean difference of 0.001759814. The group 1.6 included *Coriolopsis caperata LDCMY42* from LDC Campus and it was supported by 99% bootstrap with the mean difference of 0.003519628.

Clade 2: Hymenochaetales - the isolates categorized in this clade were found in all study sites except Thenkasi. Twelve isolates belonging to the genus *Fulvifomes*, *Phellinus* and *Inonotus* were categorised in this clade. They are *Fulvifomes fastuosus* (LDCMY39 and LDCMY43), *Inonotus rickii* (LDCMY52), *Phellinus badius* (LDCMY36) and *Phellinus* sp. (LDCMY23, LDCMY24, LDCMY28, LDCMY34 and LDCMY45). Molecular phylogeny analysis confirmed that two strains (LDCMY39 and LDCMY43) obtained from Lady Doak College campus as *Fulvifomes fastuosus*. The isolates showed highest similarity with the strains reported from Sri Lanka (KR867653) and South Korea (AY558615) and supported with 95% bootstrapping. The host for both the strains were *Albizzia* sp. We further provided the first significant report on more precise identification of *Fulvifomes fastuosus* on the basis of the genetic information. A strain collected from Ayyanar falls was identified as *Inonotus rickki* (LDCMY52) that shared 100% similarity with the strains previously reported from India. The genus *Phellinus* was found to be present in all study sites. *Phellinus badius* LDCMY36 shared 93% relatedness with the strain CBS 449.76 from South Korea. This was the first molecular evidence of the species *Phellinus badius* from India.

This Clade 2 was supported by 100% bootstrap value and consisted of 4 groups (2.1–2.4). The Group 2.1 includes *Fulvifomes fastuosus* (95% bootstrap) with the mean difference of 0.082737938; Group 2.2 was supported by 94% bootstrap and includes *Phellinus sp*. (0.100297219); Group 2.3 has only *Inonotus rickki* and supported by 100% bootstrap value and the mean difference was 0.27677544. *Phellinus badius* (99% bootstrap) along with few strains of *Phellinus sp*. were categorised in Group 2.4. The mean difference within the group was 0.096520676.

Clade 3: Russales - This group consisted of samples collected only from Tirunelveli and supported by 100% bootstrap value and consisted of 2 groups (3.1 & 3.2). Two strains (LDCMY57 and LDCMY58) supported with 93% bootstrap value and identified as *Amylosporus sp*. belonging to the family Bondarzewiaceae and grouped in 3.2. The mean difference among the isolates in this group was 0.134112602. These isolates showed similarity with the strains reported from India (BAB-5055 and BAB-5255), China (Dai 7803) and USA (JV080620J).

The morphological and culture characteristics of first time reported strains from India *Ganoderma wiiroense* and *Fulvifomes fastuosus* along with *Phellinus badius* are given below.

### ***Ganoderma wiiroense***

**Figure 8:**
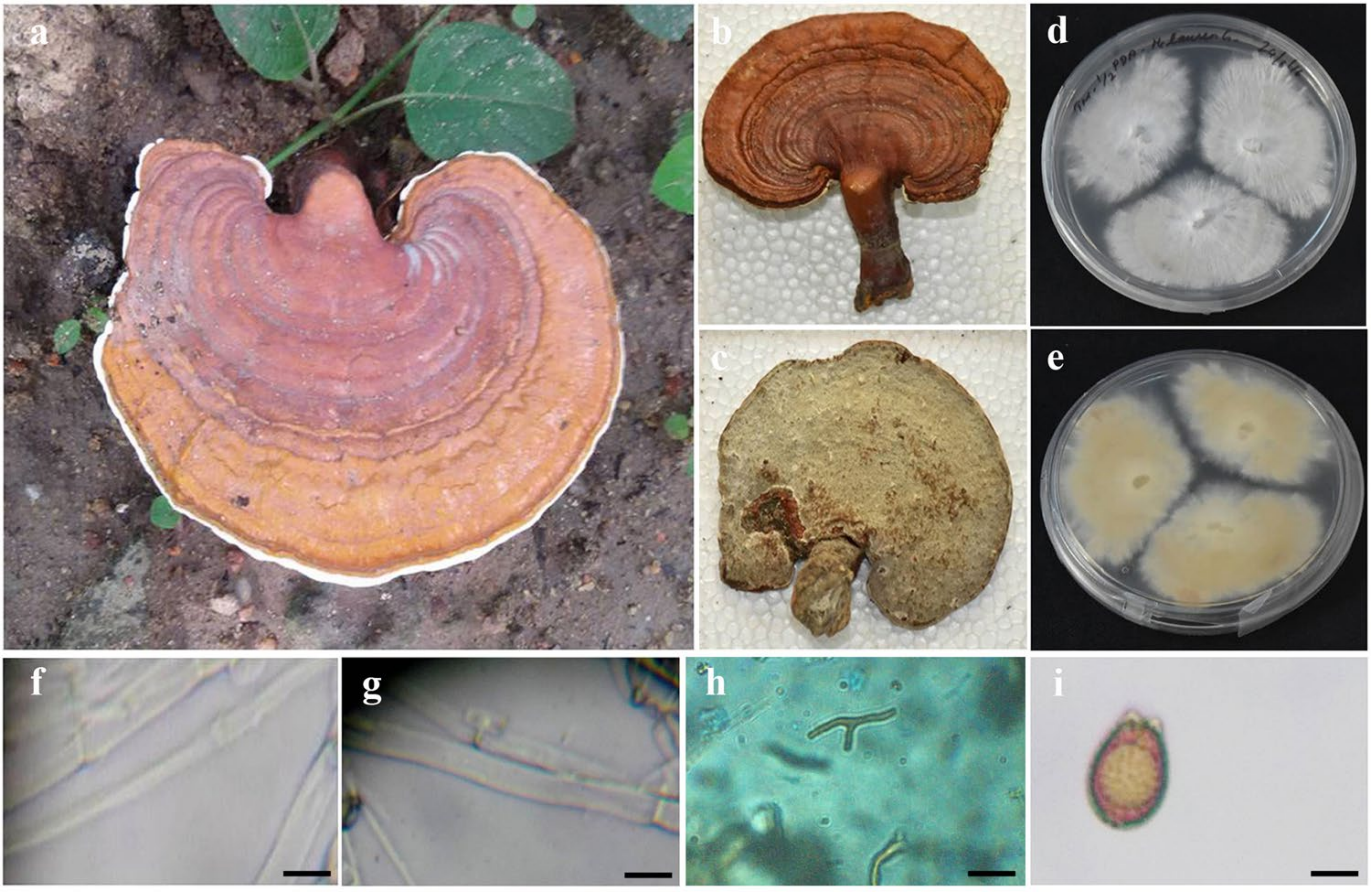
Morphology of *Ganoderma wiiroense:* (**a**) Basidiomata; (**b**) Pileal surface; (**c**) Hymenial surface; (**d** and **e**) Pure culture; (**f**) Skeletal Hyphae; (**g**) Generative hyphae; (**h**) Binding hyphae; (**i**) Basidiospores. (Scale: 20X – h; 40X – f and G; 100X – i).

Annual, pileate, basidiocarp, sessile, woody hard, white to creamy yellow when dry. Size of the pileus 10.5 cm × 7.5 cm; Hymenophore poroid, Hyphal system trimitic, generative hyphae with clamp connections, hyaline, thin-walled, branched, 2–4 µm in diameter; skeletal hyphae occasionally branched, 2.5–7.5 μm thick; binding and skeleton-binding hyphae hyaline. Spores ellipsoid (Fig. 8). Colonies of *G*. *wiiroense* on PDA was fast growing, 22–37 mm diameter after 3 days and took 7 days to completely colonize 80 mm diameter plates.

### ***Fulvifomes fastuosus***

**Figure 9:**
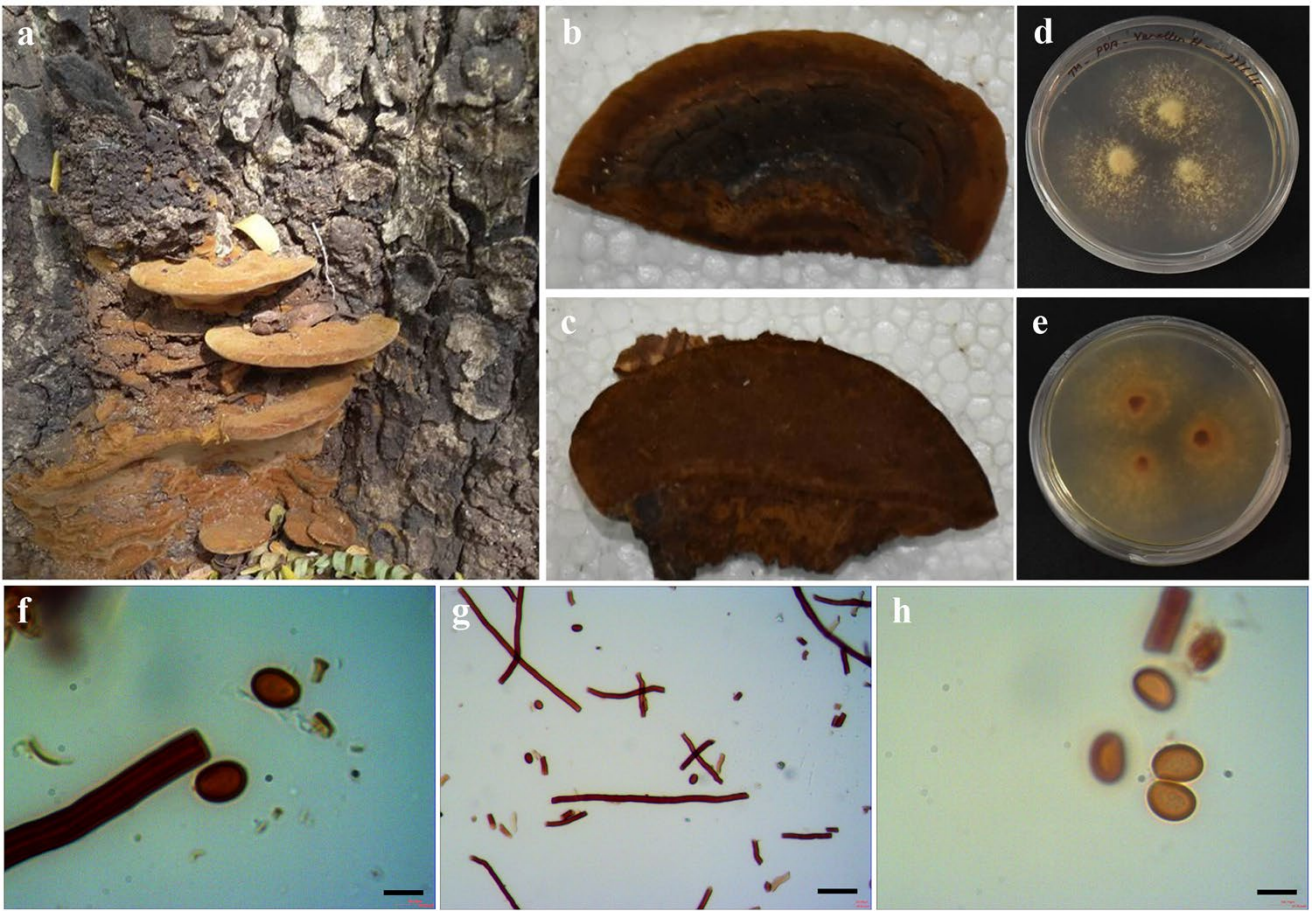
Morphology of *Fulvifomes fastuosus*: (**a**) Basidiomata attached to the host; (**b**) Pileal surface; (**c**) Hymenial surface; (**d**,**e**) Pure culture; (**f**) Skeletal Hyphae; (**g**) Generative hyphae; (**h**) Basidiospores. (Scale: 40X– g; 100X– f & h).

Perennial, pileate, basidiocarp, sessile, woody hard and without odour or taste when dry. Size of the pileus 4.5 cm × 2 cm; Hymenophore poroid, hyphal system Dimitic; generative hyphae without clamp connections, hyaline, thin-walled, simple septate, occasionally branched, 2–3 µm in diameter; skeletal hyphae thick-walled with broad lumen, unbranched, 3–5 µm in diameter. Tissue darkening in KOH. Hymenial setae absent. Spores: subglobose, yellowish, thick-walled, smooth 3.4–5.7 × 3.1–4.2 μm. Yellowish brown, dark reddish brown in KOH (Fig. 9). Colonies of *Fulvifomes fastuosus* on PDA plate was slow compared to *Ganoderma* strains, 25–28 mm diameter after 7 days and took 20 days to completely colonize 80 mm diameter plates.

### ***Phellinus badius***

**Figure 10:**
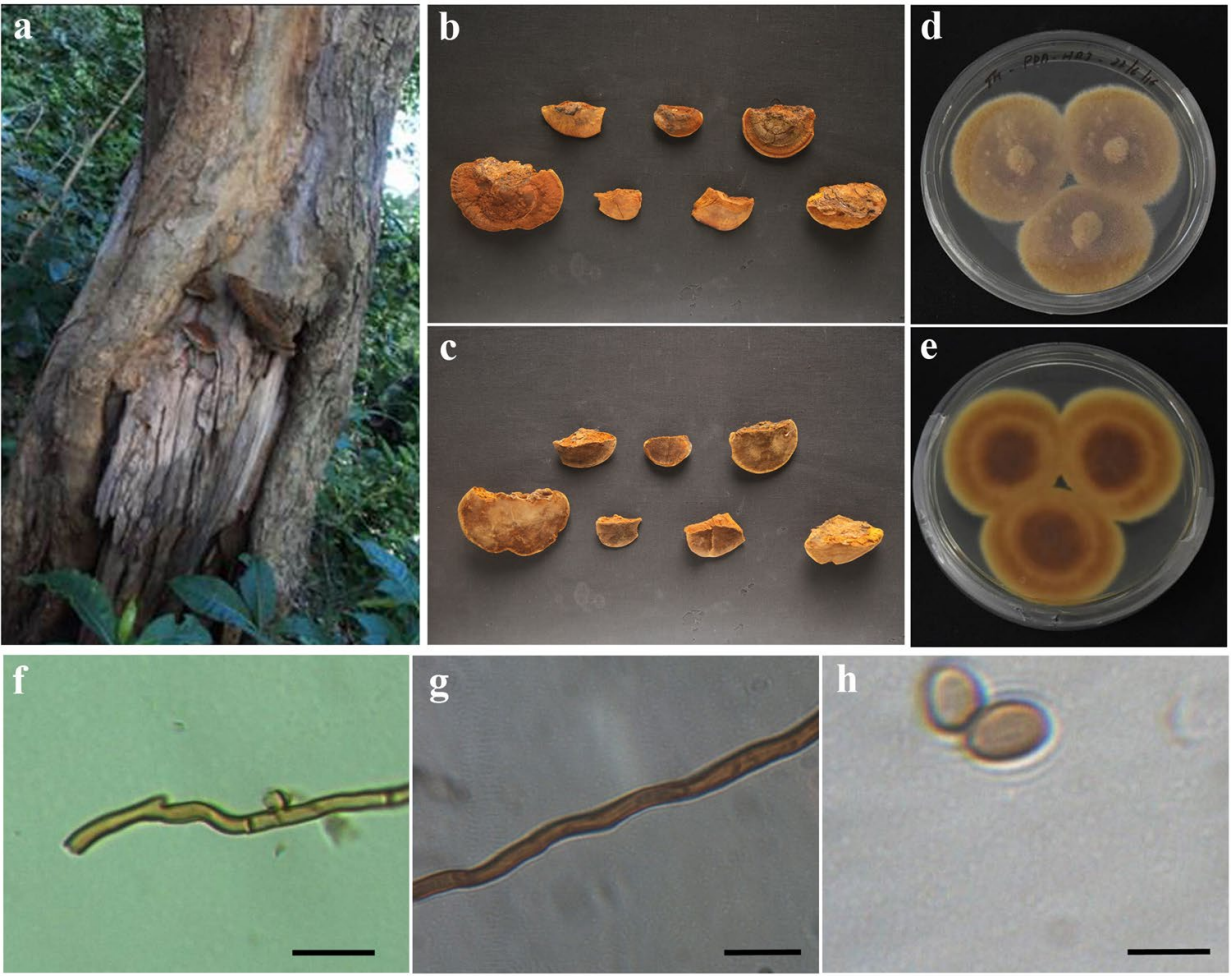
Morphology of *Phellinus badius*. (**a**) Basidiomata attached to the host; (**b**) Pileal surface; (**c**) Hymenial surface; (**d**,**e**) Pure culture; (**f**) Skeletal Hyphae; (**g**) Generative hyphae; (**h**) Basidiospores. (Scale: 40X– f & g; 100X– h).

Perennial, pileate, basidiocarp, sessile, woody hard, easily detachable from the host. Hymenophore poroid, hyphal system dimitic; generative hyphae thin walled, simple septate, clampless, moderately branched, hyaline to pale yellow, 3.47 µm; skeletal hyphae thick walled (4.35 µm); Hymenial setae absent. Spores: ellipsoid, moderately thick walled, 4.21–5.54 × 2.83–4.13 μm. Yellowish brown, dark reddish brown in KOH (Fig. 10). The growth of *Phellinus badius* on PDA was slow, 23–24 mm diameter after 7 days and took 15 days to completely colonize 80 mm diameter plates.

## Discussion

Fungi are ubiquitous in nature and distributed in all ecosystem. It can survive in diversified habitats such as air, water, soil, litter etc. It contains 1.5 million species, of which 74,000 species are named^4^. The phylum basidiomycota consist of 37% of all described fungal species^33^. Threats to fungi due to habitat destruction are a global concern as they play an important role in human welfare^19^. To understand the distribution and diversity of macrofungi in South India, the basidiomata were collected from living trees, wood log and leaf litters during the rainy season (November to January).

The *Basidiomycetes* were usually classified based on phenotypic traits; however, classification based on morphological characteristic features alone will be flawed and misleading and the use of molecular classification was found to be more reliable^34,35^. So far, only 5% of fungal strains were isolated as pure cultures and several described species were acknowledged only as herbarium specimens^19^. In the present study, pure culture (Fig. 6) was raised from 49% of the isolates and the molecular data were obtained for 65% of the isolates. These molecular data helped in identification of the isolates and was used for construction of genetic diversity among the macrofungal isolates.

### Molecular phylogeny of the macrofungal isolates

The molecular systematics of macrofungi has been studied by various methods using DNA-DNA hybridization, restriction enzyme analysis - RFLP, rDNA, mtDNA and sequencing analysis of ITS^27^. Pectinase isoenzyme^36^, manganese superoxide dismutase^37,38^, ITS and 25S ribosomal sequences^34,35,39^ were used to construct molecular phylogeny in macrofungal species. Later, ITS was used as a DNA barcode for fungal identification^32,40,41^. In this study, amplification of nuclear ribosomal ITS was used to identify the isolates. The identified isolates belong to three families namely Polyporales, Hymenochaetales and Russuales. The representative strains of the Polyporales from this study were *Coriolopsis caperata*, *Fomitopsis ostreiformis*, *Ganoderma resinaceum*, *Ganoderma sp*., *Ganoderma wiiroense* and *Trametes elegans*. The isolated strains belonging to Hymenochaetales were *Fulvifomes fastuosus*, *Inonotus rickii*, *Phellinus* sp. and *Phellinus badius*. *Amylosporous* sp. was the only strain found in our study from the family Russuales. We are the first to report the occurrence of *Ganoderma wiiroense* and *Fulvifomes fastuosus* with morphological and molecular evidence; and also provided the molecular evidence for *Phellinus badius* from India.

*G*. *wiiroense* belonging to the Family Polyporales was first reported from Upper Western region of Ghana^42^. There were only 8 strains available in the GenBank for *G*. *wiiroense*, where two from Ghana^42^ and the rest from this study. Crous *et al*.^42^ reported that *G*. *lucidum* (TVK1, India; GenBank FJ982798) was closer to *G*. *wiiroense*. In our study, we also found that the *G*. *lucidum* FJ982798 was closer to *G*. *wiiroense* than any other *Ganoderma* strains reported in this study.

The genus *Phellinus* belonging to the Family Hymenochaetaceae were important owing to their medicinal values^18,43^. Three hundred and sixty-seven *Phellinus* has been reported in the CBS (http://www.punenvis.nic.in/bd_list.htm). In India, eighteen *Phellinus* species have been reported from Kerala^44,45^, *P*. *nilgheriensis* (Mont.) Cunn., *P*. *shaferi* from Gujarat^46,47^ and *P*. *badius* was described morphologically from Punjab^48^. This study provides the first report on molecular evidence for *P*. *badius* from India.

The genus *Fulvifomes* Murrill was segregated from *Phellinus* Quél., Murrill^49^ and typified with *F*. *robiniae* (Murrill). It was not accepted as a separate genus and treated as a subgenus of *Phellinus* till 1999^50^. Later, comprehensive evidences based on molecular phylogenetic analyses proved that it as an independent genus closely associated with *Aurificaria* Reid and *Phylloporia* Murrill^51,52^. The key characteristics of *Fulvifomes* are pileate basidiocarps, a dimitic hyphal system, coloured basidiospores and absence of setae^51^. Species with resupinate basidiocarps and/or hymenial setae were included into *Fulvifomes* based on morphological studies^43^. Recently, species with monomitic hyphal system were included in *Fulvifomes* by Zhou^53^.

*Fulvifomes fastuosus* was described by Bondartseva and Herrera^54^. There are 162 reports available in GenBank on the genus Fulvifomes based on molecular data and among them only 18 sequences were on *F*. *fastuosus*. The species *F*. *fastuosus* was described from China^43^, Thailand^55^ and Sri Lanka^56^. In this study based on molecular phylogeny, two strains collected from Lady Doak College, Tamilnadu, India were identified as *Fulvifomes fastuosus*.

### Macro and micromorphological characteristic features of *G. wiiroense, P. badius* and *F. fastusosus*

The identification based on molecular means has been checked with the macro- and micro-morphological characteristic features and were found to be similar with the reported strains. However, the observation on basidiospores was different from the other reports for *P*. *badius* and *F*. *fastuosus*. The basidiospores of *P*. *badius* are ovoid to subglobose to globose and 4–6 × 4–5.5 μm^44^. Singh and colleagues^48^ reported that basidiospores were broadly ellipsoid to subglobose. Our observation shows the *P*. *badius* basidiospores were ellipsoid and 4.21–5.54 × 2.83–4.13 μm. The basidiospores of *F*. *fastuosus* were subglobose, thick-walled, smooth 4.49 × 4.01 μm^56^. According to Dai^43^, the basidiospores are 5–6.1 × 4.2–5.6 μm. Our observations shows the basidiospores were 3.4–5.7 × 3.1–4.2 μm, which was smaller than Dai^43^, but similar to Ediriweera *et al*.^56^. However, the variation in the ratio (Q) was the same as previously reported of *F*. *fastuosus* strains. The variation in the size of basidiospores might be due to their geographical niche as well as depending on their nutrients from the host species.

### Host preference by the macrofungal isolates

There are several factors that influence the distribution of fungi namely ecological niche, climatic conditions, host/substrate type, distribution of fauna and flora^19^. To study host preference, basidiomata were collected from the living trees, wood log, and leaf litters. Later, the basidiomata was identified by molecular classification.

In India, the information on *Ganoderma* was first published in the early 1900s^57^. Nearly 144 hosts were recorded in India^58^. Among them coconut, betelnut, Casuarina, *Areca catechu*, *Dalbergia sissoo* and *Toona ciliata*^59,60^ was observed as obvious host of *Ganoderma* sp. In India and Sri Lanka, *Cocus nucifera* showed high incidence as a host for *Ganoderma* species^58,61–63^. From this study, it was observed that *Ganoderma* sp. grown on the following host species: *Albizzia* sp., *Tamarindus* sp., *Azadirachta* sp. and *Coccus nucifera*. *Fomitopsis ostreiformis* belonging to Ganodermataceae has the host species *Albizzia* sp., and *Coriolopsis caperata* from wood log. The newly reported *Ganoderma wiiroense* has been collected from the trees of *Albizzia* sp., (Table 1).

The species *Fulvifomes fastuosus* belongs to the family Hymenochaetaceae and reported to have medicinal properties^43^. The *F*. *fastuosus* has been reported in the trees of *Xylocarpus granatum*^55^. In this study, *F*. *fastuosus* were found in the host trees of *Albizzia* sp.

The genera *Phellinus* have wide host range. Globally *Quercus* sp. is the more susceptible host and, in India *Mangifera* sp. followed by *Acacia*, *Artocarpus* and *Albizzia* are the predominant host of *Phellinus*^64,65^. It was observed that *Albizzia* sp. is the host preferred by the genera *Phellinus*.

The genera *Amylosporus* was first reported in India among the Asian countries^66^ with bamboo as their host^67^. In this study, the *Amylosporus* sp. was found in the host *Nerium* sp. and *Albizzia* sp. Interestingly from this study, *Albizzia* sp. is found to be the host preferred by most of the macrofungal isolates. This might be due to the abundance of this species in the vicinity of the collected macrofungi.

To conclude, we have identified and report two new macrofungal species *G*. *wiiroense* and *F*. *fulvifomes* and molecular evidence for *P*. *badius* from India. It was observed that *Albizzia* sp., as the host preferred by most of the macrofungal isolates. Our data provide the existence of *G*. *wiiroense* in India; however, we were unable to trace of out the origin of how *G*. *wiiroense* might have cross boundaries. We can only speculate *G*. *wiiroense* already exists in India; because of the lack of intense mycological study prior, this is the first report on it. These data gains us insight on macrofugal diversity in India, which can be used for the prospection of macrofungi in biomedical and industrial applications.

## Methodology

### Sample Collection and culture of isolates

Fresh basidiomata of the wild mushrooms belonging to the division basidiomycota were collected from different locations in Dindigul (Ayyanar falls), Madurai (Lady Doak College Campus, Nagamalai, Pudhupatti), Coimbatore (Kovai Kutralam), Thenkasi and Tirunelveli, Tamilnadu (India) during 2013–2017 on rainy seasons i.e., November to January. The basidiomata were cleaned and aseptically transferred to the lab. After surface sterilization with 70% ethanol, small pieces from the contextual layer of basidiomata^68^ were transferred to sterile potato dextrose agar (PDA) medium supplemented with streptomycin. The plates were incubated at 37 °C for 5–7 days. The pure culture was obtained by continuous sub culturing and used for further analysis. The isolates were stored in PDA plates and slants. The basidiomata were then dehydrated with naphthalene balls for future studies.

The radial growth of the mycelium of all the isolates on the PDA medium was measured using a ruler. Five-millimetre mycelial plugs were removed from the growing edge of the 7-day-old pure culture and inoculated on to the centre of the 80 mm petriplates containing PDA. According to Tomkin^69^ and our observation, the growth is not constant in the early stage. The lag phase was shorter (1 day) in some strains and longer (5 days) in some strains. The radial/lateral expansion was measured after three days (i.e., 3^rd^ day for strains with shorter lag phase and 7^th^ day for strains with longer lag phase) in diameter (in mm), and the number of days taken to completely colonize 80 mm petridish was recorded. All the measurements were made in triplicates. The representative voucher specimens were deposited in the Department of Biotechnology, Lady Doak College, Madurai, Tamilnadu, India. Taxonomical identification of the isolates was carried out based on molecular identification methods.

After identification, the macromorphological characteristic features such as shape, color, hymenial surface of the basidiomata were studied according to published description^70^. Microscopical observations (hyphal system, presence/absence of setae and basidiospores) were carried out using brightfield microscope (Olympus system microscope model CX41). Slides were prepared using 5% KOH and cotton blue^71^.

### Molecular characterization of the isolates

#### Genomic DNA Isolation, PCR amplification and sequencing

Genomic DNA of all the isolates were extracted as described by Moncalvo *et al*.^35^. 10 mg of mycelial biomass was homogenized with 3% SDS extraction buffer (3 g SDS, 50 mM Tris, 150 mM NaCl and 80 mM Na_2_EDTA) and incubated at 60 °C for 20–30 min. The 5.8S nuclear ribosomal RNA gene was amplified using ITS1 (CTTGGTCAT TTAGAGGAAGTAA) and ITS4 (CAGGAGACTTGTACACGGTCCAG) primers^30^. PCR amplification was carried out using the following condition: initial denaturation (95 °C, 2 min), denaturation (94 °C, 45 sec), annealing (50 °C, 45 sec), extension (72 °C, 1.30 min), final extension (72 °C, 5 min). The PCR products were purified and sequenced (Chromous Biotech Pvt. Ltd, Bangalore). The sequences were read bidirectionally for both strands of the entire ITS1, 5.8S rDNA and ITS2 region. The DNA sequence obtained from both the strands was edited and contig assembly was carried out using DNA Baser sequence assembly software (V.4.36.0). The assembled sequences were submitted to GenBank Database.

#### Phylogenetic analysis

Additional ITS sequences of Basidiomycetes were downloaded from GenBank to clarify the interspecies relationship. The phylogenetic tree was constructed by maximum likelihood (ML) analysis in MEGA 6 software^72^. The tree inference options were set as follows: Heuristic Method Nearest-Neighbor-Interchange (NNI) with the very strong branch swap filter with 1000 bootstrap replicates, gaps were treated as missing.

## References

[1] Hawksworth DL (2001). The magnitude of fungal diversity: the 1.5 million species estimate revisited** Paper presented at the Asian Mycological Congress 2000 (AMC 2000), incorporating the 2nd Asia-Pacific Mycological Congress on Biodiversity and Biotechnology, and held at the University of Hong Kong on 9–13 July 2000. Mycological research.

[2] Hawksworth DL (1991). The fungal dimension of biodiversity: magnitude, significance, and conservation. Mycological research.

[3] Porras-Alfaro A (2008). Novel root fungal consortium associated with a dominant desert grass. Applied and environmental microbiology.

[4] Hawksworth, D., Kirk, P., Sutton, B. & Pegler, D. *Ainsworth and Bisby’s Dictionary of the fungi*. 8th edn, xii + 616 (CAB International, 1995).

[5] Singh SP, Pande K, Upadhyay VP, Singh JS (1990). Fungal communities associated with the decomposition of a common leaf litter (Quercus leucotrichophora A. Camus) along an elevational transect in the Central Himalaya. Biology and Fertility of Soils.

[6] Watling R (1997). Pulling the threads together: habitat diversity. Biodiversity & Conservation.

[7] Lodge, D. J., Hawksworth, D. L. & Ritchie, B. J. In *Biodiversity and Ecosystem Processes in Tropical Forests* (eds Orians, G. H., Dirzo, R. & Cushman, J. H.) 69–100 (Springer Berlin Heidelberg, 1996).

[8] Wilkins WH, Ellis EM, Harley JL (1937). The ecology of the larger fungi: Constancy and frequency of fungal species in relation to certain vegetation communities, particularly Oak and Beech. Annals of Applied Biology.

[9] Tóth BB, Barta Z (2010). Ecological studies of ectomycorrhizal fungi: an analysis of survey methods. Fungal Diversity.

[10] Wiensczyk, A. M., Gamiet, S., Durall, D. M., Jones, M. D. & Simard, S. W. Ectomycorrhizae and forestry in British Columbia: A summary of current research and conservation strategies. *Journal of Ecosystems and Management* **2** (2002).

[11] Avis PG, Gaswick WC, Tonkovich GS, Leacock PR (2017). Monitoring fungi in ecological restorations of coastal Indiana, USA. Restoration Ecology.

[12] van Dijk H, Onguene NA, Kuyper TW (2003). Knowledge and Utilization of Edible Mushrooms by Local Populations of the Rain Forest of South Cameroon. AMBIO: A Journal of the Human Environment.

[13] Enow E, Kinge TR, Tabi EM, Thiobal N, Mih AM (2013). Diversity and distribution of macrofungi (mushrooms) in the Mount Cameroon Region. Journal of Ecology and The Natural Environment.

[14] Ramsbottom, J. *Mushrooms and toadstools: A study of the activities of fungi* (Collins 1953).

[15] Mizuno T (1995). Bioactive biomolecules of mushrooms: Food function and medicinal effect of mushroom fungi. Food Reviews International.

[16] Wasser S (2002). Medicinal mushrooms as a source of antitumor and immunomodulating polysaccharides. Applied microbiology and biotechnology.

[17] Wasser, S. P. & Weis, A. L. Medicinal properties of substances occurring in higher basidiomycetes mushrooms: current perspectives. *International Journal of medicinal mushrooms* **1** (1999). 9987601

[18] Hwang BS, Lee IK, Choi HJ, Yun BS (2015). Anti-influenza activities of polyphenols from the medicinal mushroom Phellinus baumii. Bioorganic & medicinal chemistry letters.

[19] Manoharachary, C. *et al*. Fungal biodiversity: distribution, conservation and prospecting of fungi from India. *Current Science*, 58–71 (2005).

[20] Natarajan K (1995). Mushroom flora of south India (except Kerala). Advances in Horticulture.

[21] Gilbertson, R. L. & Ryvarden, L. *North American Polypores: Megasporoporia-Wrightoporia* (Fungiflora, 1987).

[22] Schmit JP, Lodge DJ (2005). Classical methods and modern analysis for studying fungal diversity. Mycology Series.

[23] Lodge, D. J. *et al*. *Terrestrial and lignicolous macrofungi* (Elsevier Inc., 2004).

[24] Genej GJ, Stchigel AM (1999). Developments in fungal taxonomy. Clinical microbiology reviews.

[25] Sugiyama J (1998). Relatedness, phylogeny, and evolution of the fungi. Mycoscience.

[26] Bridge P, Hawksworth D (1990). New horizons in the biosystematics of filamentous fungi. Genetic Engineer and Biotechnologist.

[27] Bruns TD, White TJ (1991). & W., T. J. Fungal Molecular Systematics. Annual Review of Ecology and Systematics.

[28] Seifert KA (2009). Progress towards DNA barcoding of fungi. Molecular Ecology Resources.

[29] Hillis DM, Dixon MT (1991). Ribosomal DNA: molecular evolution and phylogenetic inference. The Quarterly review of biology.

[30] White, T. J., Bruns, T., Lee, S. & Taylor, J. In *PCR Protocols: A Guide to* Methods *and Applications* (eds Innis, M. A., Gelfand, D. H., Shinsky, J. J. & White, T. J.) 315–322 (Academic Press, 1990).

[31] Gardes M, Bruns TD (1993). ITS primers with enhanced specificity for basidiomycetes - application to the identification of mycorrhizae and rusts. Molecular ecology.

[32] Schoch CL (2012). Nuclear ribosomal internal transcribed spacer (ITS) region as a universal DNA barcode marker for Fungi. Proceedings of the National Academy of Sciences.

[33] Kirk, P. M., Cannon, P. F., David, J. & Stalpers, J. A. *Ainsworth and Bisby’s dictionary of the fungi*. (CABI publishing, 2001).

[34] Moncalvo JM, Wang HF, Hseu RS (1995). Gene phylogeny of the Ganoderma lucidum complex based on ribosomal DNA sequences. Comparison with traditional taxonomic characters. Mycological Research.

[35] Moncalvo JM, Wang HH, Hseu RS (1995). Phylogenetic Relationships in Ganoderma Inferred from the Internal Transcribed Spacers and 25S Ribosomal DNA Sequences. Mycologia.

[36] Miller RNG (1995). Isozyme analysis for characterization of Ganoderma strains from south-eastAsia 1. EPPO Bulletin.

[37] Pan SM, Ye JS, Hseu RS (1997). Purification and characterization of manganese superoxide dismutase from Ganoderma microsporum. Biochemistry and molecular biology international.

[38] Frealle E (2005). Manganese superoxide dismutase in pathogenic fungi: an issue with pathophysiological and phylogenetic involvements. FEMS immunology and medical microbiology.

[39] Moncalvo, J. M. In *Gano*derma. D*is*eases *of perennial crops* (eds Flood, J., Bridge, P. D. & Holderness, M.) Ch. 2, 23–45 (CABI, 2000).

[40] Vancov T, Keen B (2009). Amplification of soil fungal community DNA using the ITS86F and ITS4 primers. FEMS microbiology letters.

[41] Iwen PC, Hinrichs SH, Rupp ME (2002). Utilization of the internal transcribed spacer regions as molecular targets to detect and identify human fungal pathogens. Medical mycology.

[42] Crous PW (2015). Fungal Planet description sheets: 371–399. Persoonia.

[43] Dai Y-C (2010). Hymenochaetaceae (Basidiomycota) in China. Fungal Diversity.

[44] Leelavathy, K. & Ganesh, P. *Polypores of Kerala* (Daya Publishing House, 2000).

[45] Ganesh P, Leelavathy K (1986). New records of Phellinus from India. Current Science.

[46] Nagadesi P, Arya A (2012). New records of lignicolous fungi deteriorating wood in India. Mycosphere.

[47] Arya, A. In *Vistas in Palaeobotany and Plant Morphology: Evolutionary and Environmental Perspectives* (eds Pant, D. D. & Srivastava, P. C.) 321–327 (UP Offset, 2004).

[48] Singh, A. P., Kaur, G. & Dhingra, G. S. In *8th International Conference on Mushroom Biology and Mushroom Products*. 83–91 (World Society for Mushroom Biology and Mushroom Products).

[49] Murrill, W. *Northern polypores*. (The Author, 1914).

[50] Dai, Y.-C. *Phellinus sensu lato (Aphyllophorales*, *Hymenochaetaceae) in East Asia*. 115 (Finnish Zoological and Botanical Publishing Board 1999).

[51] Wagner T, Fischer M (2002). Proceedings towards a natural classification of the worldwide taxa Phellinus s.l. and Inonotus s.l., and phylogenetic relationships of allied genera. Mycologia.

[52] Larsson KH (2006). Hymenochaetales: a molecular phylogeny for the hymenochaetoid clade. Mycologia.

[53] Zhou L-W (2014). Notes on the taxonomic positions of some Hymenochaetaceae (Basidiomycota) species with colored basidiospores. Phytotaxa.

[54] Bondartseva MA, Herrera S, Sandoval D, Cejas F (1992). Taxonomical problems of the Cuban Hymenochaetaceous fungi. Mikol Fitopatol.

[55] Sakayaroj J (2012). Molecular characterization of basidiomycetes associated with the decayed mangrove tree Xylocarpus granatum in Thailand. Fungal Diversity.

[56] Ediriweera, S., Wijesundera, R., Nanayakkara, C. & Weerasena, O. A new record of Fulvifomes fastuosus from Sri Lanka. *Journal of the National Science Foundation of Sri Lanka* **42** (2014).

[57] Lloyd, C. G. *Mycological notes 1–75*. Vol. 1–1364 (CG Lloyd, 1895–1925).

[58] Sankaran KV, Bridge PD, Gokulapalan C (2005). Ganoderma diseases of perennial crops in India – an overview. Mycopathologia.

[59] Butler, E. J. *Some Disease of Palms*. Vol. 1, 299–310 (Thacker, Spink & Company, 1906).

[60] Butler E (1909). Fomes lucidus (Leys) Fr., a suspected parasite. Indian Forester.

[61] Snehalatharani A, Maheswarappa H, Devappa V, Malhotra S (2016). Status of coconut basal stem rot disease in India–A review. Indian Journal of Agricultural Sciences.

[62] Peries O (1974). Ganoderma basal stem rot of coconut: a new record of the disease in Sri Lanka. Plant Disease Reporter.

[63] Petch, T. & Bisby, G. R. The fungi of Ceylon *Peradeniya Manual*. **6** (1950).

[64] Ranadive K, Jagtap N, Vaidya J (2012). Host diversity of genus Phellinus from world. *Elixir Appl*. Botany.

[65] Ranadive K (2012). Host Distribution of Phellinus from India. Indian Journal of Forestry.

[66] Roy, A. & De, A. B. *Polyporaceae Of India*. (International Book Distributors, 1996).

[67] Tarafder E (2017). Contribution to the Macromycetes of West Bengal, India: 13–17. Research Journal of Pharmacy and Technology.

[68] Lodge, D. J., Ammirati, J. F., O’Dell, T. E. & Mueller, G. M. In *Biodiversity of fungi: inventory and monitoring methods* (eds Mueller, G. M., Bills, G. & Foster, M. S.) 128–158 (Elsevier Academic. San Diego, California, 2004).

[69] Tomkins RG (1932). Measuring growth: The petri dish method. Transactions of the British Mycological Society.

[70] Kornerup, A. & Wanscher, J. H. *Methuen Handbook of Colour*. 3 edn, (E. Methuen, 1978).

[71] Ryvarden, L. *Genera of polypores: nomenclature and taxonomy*. 1–373 (Lubrecht & Cramer Ltd, 1991).

[72] Tamura K, Stecher G, Peterson D, Filipski A, Kumar S (2013). MEGA6: Molecular Evolutionary Genetics Analysis version 6.0. Mol Biol Evol.

